# The NIH-NIAID Filariasis Research Reagent Resource Center

**DOI:** 10.1371/journal.pntd.0001261

**Published:** 2011-11-29

**Authors:** Michelle L. Michalski, Kathryn G. Griffiths, Steven A. Williams, Ray M. Kaplan, Andrew R. Moorhead

**Affiliations:** 1 Department of Biology and Microbiology, University of Wisconsin Oshkosh, Oshkosh, Wisconsin, United States of America; 2 Department of Biological Science, Smith College, Northampton, Massachusetts, United States of America; 3 Department of Infectious Disease, College of Veterinary Medicine, University of Georgia, Athens, Georgia, United States of America; Biomedical Research Institute, United States of America

## Abstract

Filarial worms cause a variety of tropical diseases in humans; however, they are difficult to study because they have complex life cycles that require arthropod intermediate hosts and mammalian definitive hosts. Research efforts in industrialized countries are further complicated by the fact that some filarial nematodes that cause disease in humans are restricted in host specificity to humans alone. This potentially makes the commitment to research difficult, expensive, and restrictive. Over 40 years ago, the United States National Institutes of Health–National Institute of Allergy and Infectious Diseases (NIH-NIAID) established a resource from which investigators could obtain various filarial parasite species and life cycle stages without having to expend the effort and funds necessary to maintain the entire life cycles in their own laboratories. This centralized resource (The Filariasis Research Reagent Resource Center, or FR3) translated into cost savings to both NIH-NIAID and to principal investigators by freeing up personnel costs on grants and allowing investigators to divert more funds to targeted research goals. Many investigators, especially those new to the field of tropical medicine, are unaware of the scope of materials and support provided by the FR3. This review is intended to provide a short history of the contract, brief descriptions of the fiilarial species and molecular resources provided, and an estimate of the impact the resource has had on the research community, and describes some new additions and potential benefits the resource center might have for the ever-changing research interests of investigators.

## History of the FR3 and Its Parasite Strains

The United States National Institutes of Health–National Institute of Allergy and Infectious Diseases (NIH-NIAID) Filariasis Research Reagent Resource Center (FR3) started in 1969 when Dr. Paul Thompson, professor and later head of the Department of Parasitology at the University of Georgia College of Veterinary Medicine (Athens, Georgia), obtained an NIH contract to establish the Filariasis Chemotherapy and Repository Research Services. Thompson, the former head of the Antiparasitic Drug Division at Parke Davis Corporation in Ann Arbor, Michigan, specialized in antimalarial drug testing using *Plasmodium berghei* as a model. He initially obtained a US Army contract to perform antimalarial drug screening, and concurrently procured an NIH grant to study immune mechanisms and immunizing agents in filariasis and NIH contract funds to establish a filariasis repository that would function to supply worms for his filariasis chemotherapy studies and for other filariasis researchers. In 1969, Drs. Hyong-Sun Ah and John Hibbard joined Thompson's filariasis immunology and repository projects. The following year, he hired entomologist Dr. John W. McCall to run the repository, because McCall had extensive experience in maintaining various mosquito species for malaria studies. Later that year, Dr. Tom Klei joined the filariasis program as an NIH postdoctoral fellow. At that time, the repository maintained two filarial species: *Litomosoides sigmodontis* (then *L. carinii*), vectored by the tropical rat mite *Ornithonyssus bacoti*; and *Acanthocheilonema* (then *Dipetalonema*) *viteae*, vectored by the argasid tick *Ornithodoros tartakovskyi*. Thompson and McCall became interested in the use of the *Brugia* system for antifilarial compound screening when Ash and Riley (University of California Los Angeles [UCLA]) published the experimental maintenance of *Brugia malayi* and *Brugia pahangi* in Mongolian jirds (*Meriones unguiculatus*) [1], [2], which are commonly known as gerbils. McCall obtained *B. pahangi*–infected dogs and *B. malayi*–infected cats from Ash's laboratory to maintain the parasites for the repository, and shortly thereafter obtained local *Dirofilaria immitis*–infected dogs, bringing the total number of filarial species housed at the University of Georgia (UGA) facility to five.

**Figure 1:**
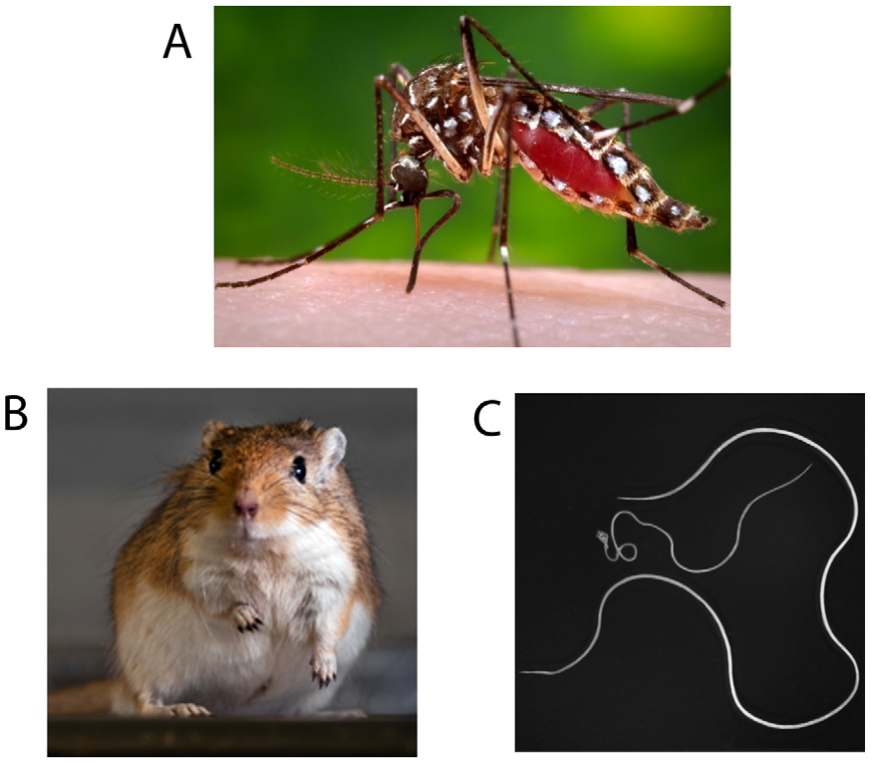
Propagation of the *Brugia malayi* life cycle. (A) *Aedes aegypti* black-eyed Liverpool strain (CDC Image Bank). (B) Mongolian gerbil (*Meriones unguiculatus*) (courtesy of Robert Storey). (C) *Brugia malayi* adults, 6.3× (courtesy of Dr. Shelly Michalski). The adult male worm is considerably smaller than the female and is identified by the curved posterior end containing spicules and associated mating structures.

In the early days of the FR3, one section of the repository contract was devoted to contract-related research. The focus of this component was protocol refinement and development, and resulted in the birth of many standard experimental filariasis protocols used today [3]–[7]. At the time in the late 1960s, the main laboratory hosts used for filarial species were primates, domestic dogs, and domestic cats; and filariasis research was limited to relatively few labs in Japan, Malaysia, the United Kingdom, and the US. [8]–[11]. The discovery of gerbil susceptibility to both *Brugia* species by Ash and Riley [1], [2], and the subsequent development of the intraperitoneal route of infection by McCall [5], had a tremendous impact on lymphatic filariasis research worldwide, and made possible the vast number of studies that have clarified our understanding of the biology, pathogenesis, and chemotherapy of filarial infections of humans and other animals. Another major accomplishment at that time was acquiring a suitable vector species that was susceptible for both *Brugia* species and for *D. immitis*. Many different vectors were tested at UGA, including local mosquitoes; however, acquisition of the black-eyed Liverpool strain (LVP) of *Aedes aegypti* developed by MacDonald [12], [13] was a major accomplishment that allowed the FR3 to propagate these parasites and supply researchers in the US and abroad with ample worm material to perform their experiments (Figure 1).

**Figure 2:**
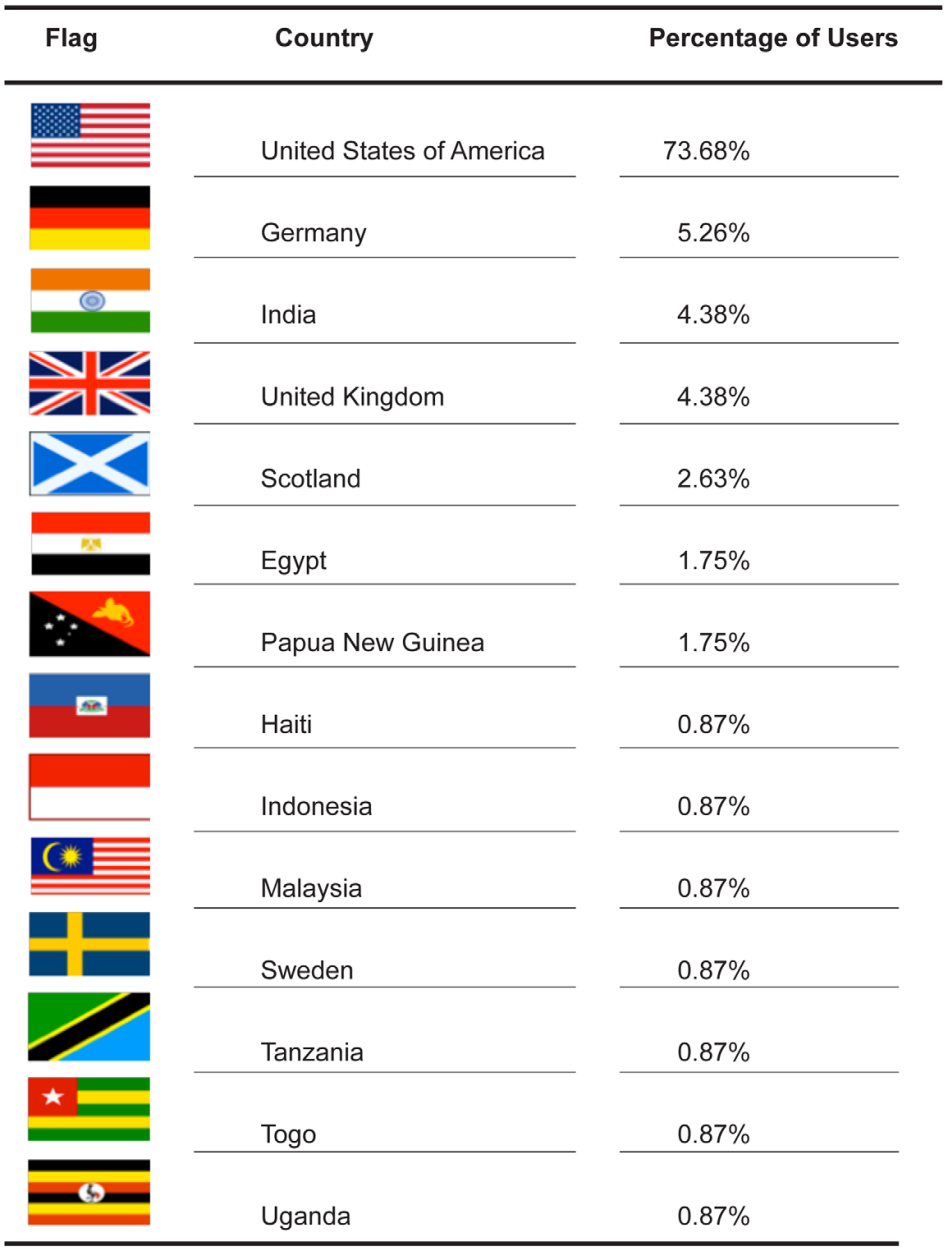
The geographic locations of recipient laboratories for FR3 parasite reagents. Country flags obtained from http://www.public-domain-image.com and http://www.clker.com.

After Thompson's death in 1973, McCall assumed the position of principal investigator of the Filariasis Repository contract. Shortly thereafter he partnered with the World Health Organization (WHO) Special Programme for Research and Training in Tropical Diseases to conduct large-scale antifilarial drug screening using several gerbil models. These studies were aimed at finding compounds with adulticidal activity, and, enabled by the large-scale production of filarial worms by the FR3, lasted for 25 years. The model organisms used in compound screening changed over the years, from gerbils with *L. sigmodontis* infections in the pleural cavity, to those with combined *L. sigmodontis* and *B. pahangi* intraperitoneal infections; to intraperitoneal *B. pahangi* combined with subcutaneous *A. viteae*; and finally single intraperitoneal *B. pahangi* infections. During that time all five filarial species were available to filariasis researchers through the Filariasis Repository. In the later years of the WHO-funded efforts (circa 2000), the Filariasis Repository ceased production of *L. sigmodontis* and *A. viteae* because they fell out of vogue for drug testing and because researcher demands had changed predominately to *B. pahangi*, *B. malayi,* and *D. immitis*. Much of McCall's efforts in the first 20 years of heading the FR3 involved protocol development and assisting principal investigators with experimental design and choosing appropriate experimental models for their research (Box 1). He estimates that in his 33-year run as principal investigator of the FR3, approximately 20–25 different academic research labs used the parasite service at any one time, for a total of hundreds throughout 14 countries (Figure 2).

**Box 1. Testimonials**

- “FR3 has been and continues to be the lifeline for filariasis research in our laboratory. Friendly staff and international experts make this a unique support service for filariasis community. My collaborators in India and Malaysia also significantly benefit out of the excellent service provided by the FR3.” Ramaswamy Kalyanasundaram, University of Illinois College of Medicine at Rockford
- “Extremely vital service; without such service our studies would have been impossible, and thus the development of novel molecular tools for post-genomic research would have been hindered.” Sara Lustigman, New York Blood Center
- “Without FR3, we would not be able to do research on filariasis. It is essential that a central resource of high quality, reliable, and consistent material be available. The cost of duplicating this resource all the labs which use it would be staggering.” Timothy Geary, McGill University
- “The life cycle is complicated and it would not be feasible to maintain the vectors and the rodent models in Canada. From what I remember of FR3, there is a real ‘art’ to maintaining these life cycles, an expertise that might take a lifetime to obtain. FR3 is the best.” Bernadette Ardelli, Brandon University
- “The capacity of the FR3 to provide unique parasite resources is CRITICAL to my work. My NIH-funded and other research would not be possible without the support of the FR3.” Michael Kron, Medical College of Wisconsin

The filarial service changed names a number of times over the years, but eventually became known as “the FR3” (Filariasis Research Reagent Resource Center), and the current contract is funded under the NIH NIAID Animal Models of Infectious Disease program (Box 2). For many filariasis investigators, the FR3 is synonymous with McCall and his efficient team of research technicians who tirelessly trained filariasis researchers in experimental maintenance of filarial worms, and facilitated hundreds of shipments of filariasis materials to academic researchers worldwide. That team included Mrs. Jung Ja Jun, who worked at the FR3 for 33 years; and Drs. Mike Dzimianski and Prasit Supakorndej, who remain at the FR3 with 28 and 25 years of experience, respectively. In 1980, when avermectins were being tested as potential anthelmintics, McCall embarked on a private venture (TRS Labs, Athens, GA) to conduct large-scale anti-filarial drug screening that was not possible at UGA due to space constraints. McCall retired from the FR3 in 2006, and oversees ∼30 projects per year mainly on chemotherapy of canine heartworm, fleas, ticks, and gastrointestinal parasites at TRS. TRS also supplies all five species of filarial worms and their vectors at cost for filariasis researchers. After McCall's retirement, the role of project director of the FR3 was taken on by Dr. Ray Kaplan (Department of Infectious Diseases, UGA), who guided the transition of the contract, and in 2011 was assumed by Dr. Andrew Moorhead (Department of Infectious Diseases, UGA).

**Box 2. Key Learning Points**

- For over 40 years, NIH-NIAID has supported a filariasis resource center to provide research material, free of charge, to hundreds of principal investigators in industrialized and developing countries. Easy access to these materials has resulted in hundreds of peer-reviewed manuscripts, meeting abstracts, book chapters, and graduate theses. Investigators in academia, not-for-profit organizations, industry, and government worldwide may receive FR3 services.
- The FR3 Parasite Resources Division offers life cycle stages of three filarial species (and their vectors): *B. malayi*, *B. pahangi*, and *D. immitis.* The *Brugia* strains, correctly denoted as “FR3 strains”, were originally collected in Kuala Lumpur in the late 1950 s/early 1960 s. They are the same strains carried by TRS Laboratories.
- The FR3 Molecular Resources Division offers filarial (*Brugia* spp., *Onchocerca* spp., *W. bancrofti*) cDNA and genomic clones, as well as anti–*B. malayi Wolbachia* surface protein wBm0432 monoclonal antibody, through BEI resources. They also offer filarial RNA and DNA preparations, the version 2 *B. malayi* microarray, and serologic reagents as well as endemic serum samples, through Smith College.
- FR3 services include personalized support for experimental questions (just call us!), an upgraded website with precise information about currently available FR3 resources and helpful links to protocols, parasite-related media, and upcoming events; a free, annual, week-long workshop offering lecture and laboratory courses taught by experts in filariasis research; and an informational booth that is displayed at the annual American Society for Tropical Medicine and Hygiene meeting where researchers can meet and greet FR3 personnel and users.
- The FR3 is committed to addressing the changing needs of its users, and assesses those needs by means of an annual electronic survey. This practice has allowed us to enrich and streamline our services to best meet the demands of the research community. Continued cooperation by the filariasis community in these surveys is the key to maintaining up-to-date services.
- Filariasis Research Reagent Resource Center website: http://www.filariasiscenter.org/
- NIH NIAID Resources for Researchers website: Filariasis Research Reagent Resource Center: http://www.niaid.nih.gov/labsandresources/resources/dmid/fila/Pages/default.aspx
- NIH NIAID Resources for Researchers website: Animal Models of Infectious Disease: http://www.niaid.nih.gov/LabsAndResources/resources/dmid/animalModels/Pages/default.aspx

In 2003, filarial resource services were greatly expanded when the success of the WHO-funded Filarial Genome Project (FGP) spurred the creation of a Molecular Resources Division of the FR3. This division is based at Smith College in Northampton, Massachusetts, and provides filariasis researchers with molecular and serological reagents, and is responsible for development of new molecular reagents and tools. It is headed by Dr. Steven Williams. The most recent change to the infrastructure of the FR3 came with the addition of a new FR3 Communication Division based at the University of Wisconsin Oshkosh, headed by Dr. Shelly Michalski. The responsibilities of this division include facilitating communication between the filariasis research community and the FR3 through annual user surveys and e-mail communications, advertising of the FR3 and the FR3 minicourse at scientific meetings, contributing to the FR3 minicourse curriculum, and maintaining the FR3 website and developing it into a multifaceted resource for filariasis research and education.

The FR3 has a Scientific Advisory Committee (SAC) that annually reviews its performance and provides advice to the director and to NIAID as to the needs of the scientific community for research agents; and on prioritization of molecular, biological, and/or genomic acquisitions as they become available. Since 2003, the three scientists who have comprised the SAC are Dr. Sara Lustigman, Member and Head, Laboratory of Molecular Parasitology, New York Blood Center; Dr. Eric Ottesen, Director Lymphatic Filariasis Support Center at The Task Force for Global Health; and Dr. Thomas Unnasch, Professor in the Department of Global Health, University of South Florida. These individuals are highly accomplished and respected members of the filariasis scientific community and together have expertise in molecular biology, immunology, genomics, proteomics, transgenics, vector biology/parasitology, pathogenesis of filariasis, and filariasis elimination programs.

## Filarial and Vector Species Provided

At its inception, the FR3 maintained five filarial species, their vertebrate hosts (dogs for *D. immitis* and *B. pahangi*, cats for *B. malayi*, cotton rats and Mongolian gerbils for *L. sigmondontis*, and gerbils for *A. viteae*, *B. malayi*, and *B. pahangi*), and their vectors (the aforementioned tick and mite species for *A. viteae* and *L. sigmodontis*, respectively), and *A. aegypti* LVP strain (for *D. immitis*, *B. malayi*, and *B. pahangi*). The previous contract, spanning 2003–2010, focused mainly on the production of *D. immitis*, *B. pahangi*, and *B. malayi*. During this time period, the FR3 responded to over 2,000 requests from almost 80 researchers for parasite materials. The number of requests for parasites has remained relatively stable over the period of the contract; however, changes in user trends have been apparent, primed largely by advances in molecular techniques that have enabled more studies on the human pathogen *B. malayi*. In general, requests for *B. pahangi* and *D. immitis* have generally decreased, while those for *B. malayi* (particularly for microfilaremic blood) have increased. Keeping current with the changing needs of filariasis researchers has proven challenging; however, the circulation of an annual user survey has helped the FR3 adjust to meet user requests (e.g., increasing the number of *B. malayi*–infected cats and augmenting mosquito production to meet an overall anticipated increase in requests for the third-stage larvae of *D. immitis*, *B. malayi*, and *B. pahangi*). Most recently, requests from users conducting comparative work on the filarial *Wolbachia* endosymbiont has resulted in additional funding for the 2011–2012 contract year to re-establish the *A. viteae* life cycle at UW Oshkosh.

## Molecular Reagents

The molecular reagents collection began in 1994 when the WHO initiated the FGP and established the laboratory of Steven Williams as the resource center for cDNA and genomic libraries and clones as well as other genomic reagents. With assistance from the laboratories of Drs. Mark Blaxter (University of Edinburgh), Barton Slatko (New England Biolabs), Alan Scott (Johns Hopkins University), and Sara Lustigman (New York Blood Center), the FGP Resource Center initially distributed cDNA libraries and cDNA clones. These cDNA libraries were used in the first stage of the *B. malayi* genome project for EST sequencing and gene identification [14]. Every cDNA clone that was sequenced was stored, and over 37,000 cDNA clones are now individually available through the FR3. This set of cDNA clones was recently duplicated and sent to the Biodefense and Emerging Infections Research Resources Repository (BEI Resources, http://www.beiresources.org/) for distribution to the scientific community under a new arrangement with NIAID.

In total, 25 cDNA libraries are available through FR3 and include libraries for various stages of *B. malayi*, *Wuchereria bancrofti*, *Onchocerca volvulus*, and *Onchocerca ochengi*. Genomic libraries in bacteriophage lambda are available for *B. malayi* and *O. volvulus*, as well as a genomic BAC library that was used to map and sequence the *Wolbachia* genome of *B. malayi* [15], [16]. Using both EST and genome sequence data from the *B. malayi* genome project [17], [18] and data available from *Wolbachia, W. bancrofti*, and *O. volvulus*, a total of 18,153 oligonucleotides were synthesized for spotting on *B. malayi* microarrays. These version 2 microarrays were developed by a consortium of researchers and are currently available by request from the FR3.

**Figure 3:**
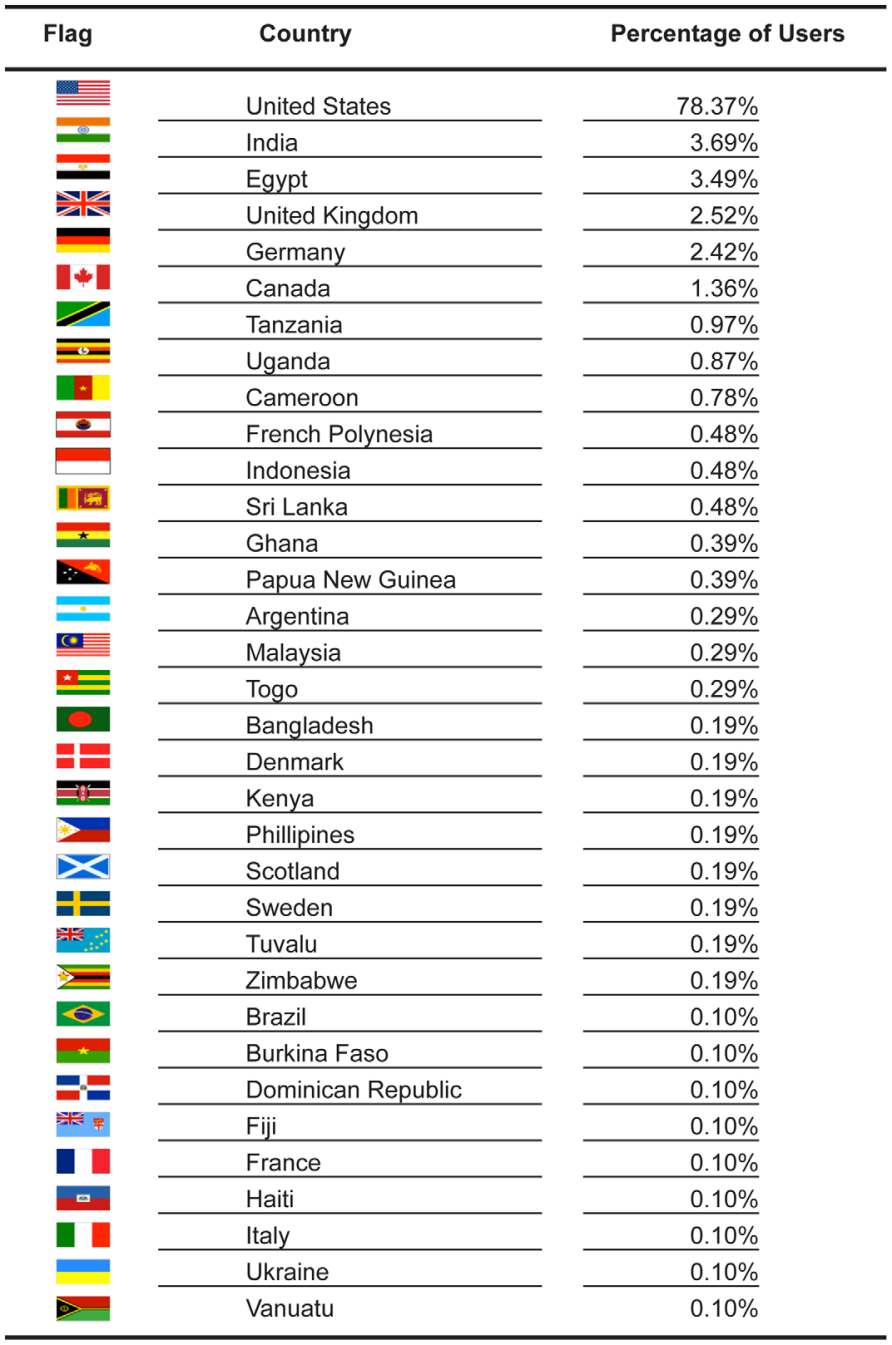
The geographic locations of recipient laboratories for FR3 molecular reagents. Country flags obtained from http://www.public-domain-image.com and http://www.clker.com.

The FR3 also has a variety of protein molecular reagents, including a serum bank with sera from infected and uninfected patients, the Bm14 antigen for serologic monitoring, and the positive control for the Binax Filariasis NOW test. The serum bank includes two separate collections: 1) the *O. volvulus* serum bank with 340 serum samples from Ecuador, Togo, Nigeria, and Cameroon, and 2) the filariasis serum bank with 363 sera from Africa, Asia, and South America. Newly available for 2011 are the monoclonal antibody mAb Bmwsp, raised against *B. malayi Wolbachia* surface protein wBm0432 [19], that is available through BEI Resources (http://www.beiresources.org/), as well as user-ready filarial RNA and DNA preparations (including whole genome-amplified *Wuchereria bancrofti* DNA) that are available through Smith College. The Molecular Division of the FR3 has proven to be a valuable resource for the filariasis research community and has to date provided researchers in 34 countries with filarial molecular reagents (Figure 3).

## Other Services: Education and Training, and the FR3 Website

In 2006, the FR3 began offering a hands-on annual minicourse to introduce students, postdoctoral fellows, principal investigators, university professors, and industry representatives to a variety of organismal and molecular techniques commonly used in filariasis research. Lectures are given daily and include topics related to vector and nematode biology, experimental models of filarial disease, FR3 molecular and parasite resources, molecular data analysis and reporting, bioinformatics, pathogenesis, and control of filarial diseases. Daily hands-on lab exercises involve vector propagation, isolation and observation of different filarial life cycle stages from vertebrate and invertebrate hosts, filarial nucleic acid purification and characterization, DNA and quantitiative RT-PCR, and host animal biology and handling. The minicourse is held at the University of Georgia (Athens, Georgia), and is free of charge; FR3 personnel and filariasis researchers teach lecture and laboratory sessions. To date, more than 50 people have participated in the minicourse, and anonymous satisfaction surveys administered on the last day of the course overwhelmingly indicate that the course met or surpassed the expectations of participants (Box 3), who enjoy the small class size, broad experience, and interaction with filariasis experts. From 2009 to 2011, the breakdown of participant type was 12% government employee, 18% faculty, 3% technician, 26% graduate students, 12% postdoctoral fellows, 6% undergraduates, 3% veterinary students, 17% industry, and 3% medical doctors. When recalling the vast number of researchers that he helped individually over the early years of the FR3, John McCall remarked that “the FR3 minicourse would have been nice then”.

**Box 3. Comments from FR3 Minicourse Participants, as Part of the Anonymous Course Evaluation**

- “A comprehensive introduction to the world of filarial parasite research. Instructive hands-on training and exciting demonstrations!”
- “It was a great experience to get to know some other members of the filarial research community.”
- “If you'd like to learn a lot about filariasis, worms, or parasitology you have to join this course!”
- “The FR3 workshop is not only very valuable for learning various aspects of filarial research, but is also very engaging, meeting all my expectations and more!”
- “This was a great all-Inclusive program that taught not only whole organism but also vector interactions and molecular biology. Anyone wanting to take a crash course in filarial worm biology and actually come out feeling like they can design their own experiments should take this minicoursel”
- “The FR3 workshop is not only very valuable for learning various aspects of filarial research, but is also very engaging, meeting all my expectations and more!”

The FR3 website (http://www.filariasiscenter.org/) offers investigators the capability of ordering resources online and accessing parasite-related and molecular protocols to facilitate their research programs. Features of the website include detailed lists of parasite and molecular reagents, online parasite and molecular resources ordering, a parasite resources inventory list that is updated monthly, information about the annual minicourse, pictures and videos submitted by the filariasis research community, helpful tips for filariasis researchers, an events calendar that displays upcoming scientific meetings, a live RSS feed that displays recent filariasis-related articles, links to filariasis/nematode/*Wolbachia*–related websites, and a filarial genomics and bioinformatics section that includes *B. malayi* genome data and microarray annotation, as well as comparative genomic information to *Caenorhabditis elegans*. The digital content of the website is continually being expanded, so that it may serve as a centralized resource for the filariasis research community.

## Impact of the Resource on Research Community

A previous publication on the NIAID Schistosomiasis Resource Center summed up its impact on the schistosomiasis research community [20] by stating “It would be impossible to give an accurate number of publications in experimental schistosomiasis that have been made possible through the use of this resource over the 40-plus years of its existence.” We echo these sentiments in regards to the FR3, because it would be impossible to count not only the published manuscripts, theses, and abstracts made possible by our services, but also the number of researchers that have obtained help with experimental design, protocol implementation, and hands-on knowledge of working with filarial nematodes and their hosts. A comprehensive search of standard literature databases reveals that over the last 3 years, approximately 68% of experimental filariasis papers are from laboratories that use FR3 services; and that over the last 4 years, approximately 50% of all filariasis presentations at the American Society for Tropical Medicine and Hygiene annual meeting acknowledged the FR3. Box 4 highlights the diversity of original studies made possible by the FR3; a complete list of peer-reviewed manuscripts that have cited the FR3 is available as Text S1, and is also on the FR3 website (http://www.filariasiscenter.org/).

**Box 4. Original Studies Made Possible by FR3 That Represent a Diversity of Disciplines (entire list available in Text S1)**

McCall JW, Malone JB, Ah H-S, Thompson PE (1973) Mongolian jirds (*Meriones unguiculatus*) infected with *Brugia pahangi* by the intraperitoneal route: A rich source of developing larvae, adult filariae, and microfilariae. J Parasitol 59: 436.

Smith HL, Rajan TV (2000) Tetracycline inhibits development of the infective-stage larvae of filarial nematodes *in vitro*. Exp Parasitol 95: 265–270.

Laney SJ, Buttaro CJ, Visconti S, Pilotte N, Ramzy RMR, Weil GJ, Williams SA (2008) A reverse transcriptase-PCR assay for detecting filarial infective larvae in mosquitoes. PLoS Negl Trop Dis 2: e251. doi:10.1371/journal.pntd.0000251

Tisch DJ, Bockarie MJ, Dimber Z, Kiniboro B, Tarongka N, Hazlett FE, Kastens W, Alpers MP, Kazura JW (2008) Mass drug administration trial to eliminate lymphatic filariasis in Papua New Guinea: changes in microfilaremia, filarial antigen, and Bm14 antibody after cessation. Am J Trop Med Hyg 78: 289–293.

Moreno Y, Nabhana JF, Solomona S, Mackenzieb CD, Geary TG. 2010. Ivermectin disrupts the function of the excretory- secretory apparatus in microfilariae of *Brugia malayi*. Proc Natl Acad Sci U S A 107: 20120–20125.

Pastrana DV, Raghavan N, FitzGerald P, Eisinger SW, Metz C, Bucala R, Schleimer RP, Bickel C, Scott AL (1998) Filarial nematode parasites secrete a homologue of the human cytokine macrophage migration inhibitory factor. Infect Immun 66: 5955–5963.

Fuhrman JA, Lane WS, Smith RF, Piessens WF, Perler FB (1992) Transmission-blocking antibodies recognize microfilarial chitinase in Brugian lymphatic filariasis. Proc Natl Acad Sci U S A 89: 1548–1552.

Raghavan N, Freedman DO, Fitzgerald PC, Unnasch TR, Ottesen EA, Nutman TB (1994) Cloning and characterization of a potentially protective chitinase-like recombinant antigen from *Wuchereria bancrofti.* Infect Immun 62: 1901–1908.

Weil GJ, Liftis F (1987) Identification and partial characterization of a parasite antigen in sera from humans infected with *Wuchereria bancrofti*. J Immunol 138: 3035–3041.

Jeffers GW, Klei TR, Enright FM, Henk WG (1987) The granulomatous inflammatory response in jirds, *Meriones unguiculatus*, to *Brugia pahangi*: an ultrastructural and histochemical comparison of the reaction in the lymphatics and peritoneal cavity. J Parasitol 73:1220–1233.

Liu LX, Weller PF (1992) Intravascular filarial parasites inhibit platelet aggregation: role of parasite-derived prostanoids. J Clin Invest 89: 1113–1120.

Samykutty A, Dakshinamoorthy G, Kalyanasundaram R (2010) Multivalent vaccine for lymphatic filariasis. Procedia Vaccinol 3: 12–18.

Blaxter ML, Raghavan N, Ghosh I, Guiliano D, Lu W, Williams SA, Slatko B, Scott AL (1996) Genes expressed in *Brugia malayi* infective third stage larvae. Mol Biochem Parasitol 77: 77–93.

Bennuru S, Semnani R, Meng Z, Ribeiro JMC, Veenstra TD, Nutman TB (2009) *Brugia malayi* excreted/secreted proteins at the host/parasite interface: stage- and gender-specific proteomic profiling. PLoS Negl Trop Dis 3: e410. doi:10.1371/journal.pntd.0000410

Ford L, Zhang J, Liu J, Hashmi S, Fuhrman JA, Oksov Y, Lustigman S (2009) Functional analysis of the cathepsin-like cysteine protease genes in adult *Brugia malayi* using RNA interference. PLoS Negl Trop Dis 3: e377. doi:10.1371/journal.pntd.0000377

Crossgrove K, Maina CV, Robinson-Rechavi M, Lochner MC (2008) Orthologues of the *Drosophila melanogaster E75* molting control gene in the filarial parasites *Brugia malayi* and *Dirofilaria immitis.* Mol Biochem Parasitol 157: 92–97.

Song C, Gallup JM, Day TA, Bartholomay LC, Kimber MJ (2010) Development of an *in vivo* RNAi protocol to investigate gene function in the filarial nematode, *Brugia malayi*. PLoS Pathog 6: e1001239. doi:10.1371/journal.ppat.1001239

Xu S, Liu C, Tzertzinis G, Ghedin E, Evans CC, Kaplan R, Unnasch TR (2011) In vivo transfection of developmentally competent *Brugia malayi* infective larvae. Int J Parasitol 41: 355–362.

Landmann F, Foster JM, Slatko B, Sullivan W (2010) Asymmetric *Wolbachia* segregation during early *Brugia malayi* embrogenesis determines is distribution in adult host tissues. PLoS Negl Trop Dis 4: e758. doi:10.1371/journal.pntd.0000758

Christensen BM, Sutherland DR. 1984. *Brugia pahangi*: exsheathment and midgut penetration in *Aedes aegypti*. Transactions of the American Microscopical Society. 103: 423–433.

Beerntsen BT, Severson DW, Christensen BM (1994) *Aedes aegypti*: characterization of a hemolymph polypeptide expressed during melanotic encapsulation of filarial worms. Exp Parasitol 79: 312–321.

## Strain Names, and Acknowledging the FR3

The FR3 provides parasite and molecular materials for only the charge of shipping to academic researchers, teaching institutions, and for-profit industrial researchers. New users must submit a registration form and completed material transfer agreements prior to receiving shipments; these documents are available on the FR3 website (http://www.filariasiscenter.org/). The *B. malayi* and *B. pahangi* strains that have been propagated at the FR3 since 1970 have been referred to by several different names (e.g., TRS, McCall, FR3); they are correctly referenced as the “FR3” strains. Both were derived from infections that Dr. John Schacher had kept in dogs and cats when he was at American University in Beirut, Lebanon, before himself relocating to UCLA in the late 1960s. Schacher originally obtained these infections from researchers in Kuala Lumpur (Larry Ash, personal communication). The *D. immitis* strain currently propagated at the FR3 was obtained from TRS labs and is denoted as the Missouri 2005 strain. When publishing work that involves the use of FR3 resources, please acknowledge the NIH/NIAID Filariasis Research Reagent Resource Center (http://www.filariasiscenter.org/) for relevant materials.

## Supporting Information

Text S1 Papers made possible by FR3.
(DOC)
