# Supplementary material for: The NIH-NIAID Filariasis Research Reagent Resource Center
Source: PLoS Negl Trop Dis. 2011 Nov 29;5(11):e1261. doi: 10.1371/journal.pntd.0001261 (PMC3226539; doi:10.1371/journal.pntd.0001261)
Supplement: Text S1 — Papers made possible by FR3. (DOC) [file pntd.0001261.s001.doc]

**Supporting Text S1 - Papers made possible by FR3**

**Current as of April, 2011**

**Animal Model/Culture**

McCall JW, Malone JB, Ah H-S, Thompson PE. 1973. Mongolian jirds (*Meriones unguiculatus*) infected with *Brugia pahangi* by the intraperitoneal route: A rich source of developing larvae, adult filariae, and microfilariae. Journal of Parasitology. 59:436. (57)

Yates JA, Schmitz KA, Nelson FK, Rajan TV. 1994. Infectivity and normal development of third stage *Brugia malayi* maintained *in vitro*. Journal of Parasitology. 80:891-894. (13)

Smith HL, Paciorkowski N, Babu S, Rajan TV. 2000. Development of a serum-free system for the *in vitro* cultivation of *Brugia malayi* infective-stage larvae. Experimental Parasitology.

95:253-264. (11)

Higazi TB, Shu L, Unnasch TR. 2004. Development and transfection of short-term primary cell cultures from *Brugia malayi*. Molecular and Biochemical Parasitology. 137:345-348. (4)

Ramesh M, McGuiness C, Rajan TV. 2005. The L3 to L4 molt of *Brugia malayi*: Real time visualization by video microscopy. Journal of Parasitology. 91:1028-1033. (2)

**Diagnosis/Treatment**

Smith HL, Rajan TV. 2000. Tetracycline inhibits development of the infective-stage larvae of filarial nematodes *in vitro*. Experimental Parasitology. 95:265-270. (46)

Rao R, Weil GJ. 2002. *In vitro* effects of antibiotics on *Brugia malayi* worm survival and reproduction. Journal of Parasitology. 88:605-611. (25)

Kanesa-thasan N, Douglas JG, Kazura JW. 1991. Diethylcarbamazine inhibits endothelial and microfilarial prostanoid metabolism *in vitro*. Molecular and Biochemical Parasitology. 49:11-20. (19)

Rajan TV. 2004. Relationship of anti-microbial activity of tetracyclines to their ability to block the L3 to L4 molt of the human filarial parasite *Brugia malayi*. American Journal of Tropical Medicine and Hygiene. 71:24-28. (19)

Klion AD, Raghavan N, Brindley PJ, Nutman TB. 1991. Cloning and characterization of a species-specific repetitive DNA sequence from *Loa loa*. Molecular and Biochemical Parasitology. 45:297-305. (15)

Laney SJ, Buttaro CJ, Visconti S, Pilotte N, Ramzy RMR, Weil GJ, Williams SA. 2008. A reverse transcriptase-PCR assay for detecting filarial infective larvae in mosquitoes. PLoS Neglected Tropical Diseases. 2:e251. (14)

Dhananjeyan MR, Milev YP, Kron MA, Nair MG. 2005. Synthesis and activity of substituted anthraquinones against a human filarial parasite, *Brugia malayi*. Journal of Medicinal Chemistry.

48:2822-2830. (10)

Tisch DJ, Bockarie MJ, Dimber Z, Kiniboro B, Tarongka N, Hazlett FE, Kastens W, Alpers MP, Kazura JW. 2008. Mass drug administration trial to eliminate lymphatic filariasis in Papua New Guinea: Changes in microfilaremia, filarial antigen, and Bm14 antibody after cessation. American Journal of Tropical Medicine and Hygiene. 78: 289–293. (10)

Cameron ML, Levy P, Nutman T, Vanamala CR, Narayanan PR, Rajan TV. 1988. Use of restriction fragment length polymorphisms (RFLPs) to distinguish between nematodes of pathogenic significance. Parasitology. 96:381-390. (9)

Rohrer WH, Esch H, Saz HJ. 1988. Neuromuscular electrophysiology of the filarial helminth *Dipetalonema vitae.* Comparative Biochemistry and Physiology Part C: Comparative Pharmacology. 91:517-523. (8)

VandeWaa EA, Bennett JL, Williams JF, Satti MZ, Geary TG. 1989. Anti-filarial effects of nine quinoline-containing drugs on adult filariae *in vitro*. Journal of Parasitology. 75:367-372. (5)

Rahman RA, Hwen-Yee C, Noordin R. 2007. Pan LF-ELISA using BmR1 and BmSXP

recombinant antigens for detection of lymphatic filariasis. Filaria Journal. 6:10 (5)

Ghedin E, Hailemariam T, DePasse JV, Zhang X, Oksov Y, Unnasch TR, Lustigman S. 2009. *Brugia malayi* gene expression in response to the targeting of the *Wolbachia* endosymbiont by tetracycline treatment. PLoS Neglected Tropical Diseases. 3:e525. (4)

Park J, Dickerson TJ, Janda KD. 2008. Major sperm protein as a diagnostic antigen for onchocerciasis. Bioorganic and Medicinal Chemistry. 16:7206-7209. (4)

Chambers EW, McClintock SK, Avery MF, King JD, Bradley MH, Schmaedick MA, Lammie PJ, Burkot TR. 2009. Xenomonitoring of *Wuchereria bancrofti* and *Dirofilaria immitis* infections in mosquitoes from American Samoa: Trapping considerations and a comparison of polymerase chain reaction assays with dissection. American Journal of Tropical Medicine and Hygiene. 80:774-781. (2)

Natarajan S, Werner C, Cameron M, Rajan TV. 1990. Isolation and characterization of a repetitive DNA element from the genome of the human filarial parasite, *Brugia malayi*. Molecular and Biochemical Parasitology. 43:39-50. (2)

Laney SL, Ramzy RMR, Helmy HH, Farid HA, Ashour AA, Weil GJ, Williams SA. 2010. Detection of *Wuchereria bancrofti* L3 larvae in mosquitoes: A reverse transcriptase PCR assay evaluating infection and infectivity. PLoS Neglected Tropical Diseases. 4:e602. (2)

Junnila A, Bohle DS, Roger Prichard R, Perepichka I, Spina C. 2007. Fluorescent

diethylcarbamazine analogues: Sites of accumulation in *Brugia malayi.* Bioconjugate Chemistry.

18: 1818-1823. (2)

Erickson SM, Fischer K, Weil GJ, Christensen BM, Fischer PU. 2009. Distribution of *Brugia malayi* larvae and DNA in vector and non-vector mosquitoes: Implications for molecular diagnostics*.* Parasites & Vectors. 2:56. (1)

Moreno Y, Nabhana JF, Solomona S, Mackenzieb CD, Geary TG. 2010. Ivermectin disrupts the function of the excretory- secretory apparatus in microfilariae of *Brugia malayi*. Proceedings of the National Acaddamy of Sciences of the United States of America. 107:20120-20125. (1)

Wenkert D, Ramirez B, Shen Y, Kron MA. 2010. *In vitro* activity of geldanamycin derivatives against *Schistosoma japonicum* and *Brugia malayi.* Journal of Parasitology Research.

2010:716498. (0)

Mahajan RS, Veerpathran A, Dakshinamoorthy G, Sharma RD, Goswami K, Reddy MV. 2010. Effect of certain antibiotics against filarial parasite *Brugia malayi in vitro*: Possible role of oxidative stress. Indian Journal of Clinical Biochemistry. 25:362-366. (0)

Licitra B, Chambers EW, Kelly R, Burkot TR. 2010. Detection of *Dirofilaria immitis* (Nematoda: Filarioidea) by polymerase chain reaction in *Aedes albopictus, Anopheles punctipennis*, and *Anopheles crucians* (Diptera: Culicidae) from Georgia, USA. Journal of Medical Entomology. 47:634-638. (0)

Tompkins JB, Stitt LE, Ardelli BF. 2010. *Brugia malayi: In vitro* effects of ivermectin and moxidectin on adults and microfilaria. Experimental Parasitology. 124:394-402. (0)

Subrahmanyam M, Belokar WK. 1979. *Wuchereria bancrofti* microfilarial antigen in the diagnosis of human filariasis by skin test. Journal of Postgraduate Medicine. 25:81-4.

Strübing U, Lucius R, Hoerafu A, Pfarr KM. 2010. Mitochondrial genes for heme-dependant respiratory chain complexes are up-regulated after depletion of *Wolbachia* from filarial nematodes. International Journal for Parasitology. 40:1193-1202. (0)

Simonsen PE, Pedersen EM, Rwegoshora RT, Malecela MN, Derua YA, Magesa SM. 2010. Lymphatic filariasis control in Tanzania: Effect of repeated mass drug administration with ivermectin and albendazole on infection and transmission. PLoS Neglegted Tropical Diseases.

4:e696. (0)

Fanning MM, Kazura JW. 1985. *Brugia malayi*: Clearance of microfilaremia induced by diethylcarbamazine independently of antibody. Experimental Parasitology. 60:396-403. (0)

Yu Z, Vodanovic-Jankovic S, Ledeboer N, Huang SX, Rajski SR, Kron M, Ben Shen B. 2011. Tirandamycins from *Streptomyces* sp. 17944 inhibiting the parasite *Brugia malayi* asparagine tRNA synthetase. Organic Letters. In print. (0)

Cho-Ngwa F, Zhu X, Metuge JA, Daggfeldt A, Grönvlk KO, Orlando R, Atwood JA, Titanjl.

2010. Identification of *in vivo* released products of *Onchocerca* with diagnostic potential, and characterization of a dominant member, the OV1CF intermediate filament. Infection, Genetics and Evolution. Article in press. (0)

Bourguinat C, Keller K, Blagburn B, Schenker R, Geary TG, Prichard RK. 2011. Correlation between loss of efficacy of macrocyclic lactone heartworm anthelmintics and P-glycoprotein genotype. Veterinary Parasitology. Article in press. (0)

**Immunology/Vaccine/Pathogenesis**

Pastrana DV, Raghavan N, FitzGerald P, Eisinger SW, Metz C, Bucala R, Schleimer RP, Bickel C, Scott AL. 1998. Filarial nematode parasites secrete a homologue of the human cytokine macrophage migration inhibitory factor. Infection and Immunity. 66:5955-5963. (122)

Chaussabel D, Semnani RT, McDowell MA, Sacks D, Sher A, Nutman TB. 2003. Unique gene expression profiles of human macrophages and dentritic cells to phylogenetically distinct parasites. Blood. 102:672-681. (119)

Fuhrman JA, Lane WS, Smith RF, Piessens WF, Perler FB. 1992. Transmission-blocking antibodies recognize microfilarial chitinase in Brugian lymphatic filariasis. Proceedings of the National Academy of Sciences of the United States of America. 89:1548-1552. (92)

Lawrence RA, Allen JE, Osborne J, Maizels RM. 1994. Adult and microfilarial stages of the filarial parasite *Brugia malayi* stimulate contrasting cytokine and Ig isotype responses in BALB/c mice. The Journal of Immunology. 153:1216-1224. (85)

Babu S, Blauvelt CP, Kumaraswami V, Nutman TB. 2006. Regulatory networks induced by live parasites impair both Th1 and Th2 pathways in patent lymphatic filariasis: Implications for parasite persistence. The Journal of Immunology. 176:3248-3256. (77)

Dimock KA, Eberhard ML, Lammie PJ. 1996. Th1-like antifilarial immune responses predominate in antigen-negative persons. Infection and Immunity. 64:2962-2967. (59)

Paciorkowski N, Porte P, Leonard D. Shultz LD, Rajan TV. 2000. B1 B lymphocytes play a critical role in host protection against lymphatic filarial parasites. The Journal of Experimental Medicine. 191:731-736. (52)

Mitre E, Taylor RT, Kubofcik J, Nutman TB. 2004. Parasite antigen-driven basophils are a major source of IL-4 in human filarial infections. The Journal of Immunology. 172:2439-2445. (51)

Li B-W, Chandrashekar R, Weil GJ. 1993. Vaccination with recombinant filarial paramyosin induces partial immunity to *Brugia malayi* infection in jirds. The Journal of Immunology.

150:1881-1885. (49)

Freedman D, Nutman TB, Ottesen EA. 1989. Protective immunity in bancroftian filariasis selective recognition of a 43-kD larval stage antigen by infection-free individuals in an endemic area. The Journal of Clinical Investigation. 83:14-22. (49)

Gnanasekar M, Rao KVN, He YX, Mishra PK, Nutman TB, Perumal Kaliraj P, Ramaswamy K.

2004. Novel phage display-based subtractive screening to identify vaccine candidates of *Brugia malayi.* Infection and Immunity. 72:4707-4715. (48)

Liu LX, Serhan CN, Weller PF. 1990. Intravascular filarial parasites elaborate cyclooxygenase- derived eicosanoids. The Journal of Experimental Medicine. 172:993-996. (46)

Raghavan N, Freedman DO, Fitzgerald PC, Unnasch TR, Ottesen EA, Nutman TB. 1994. Cloning and characterization of a potentially protective chitinase-like recombinant antigen from *Wuchereria bancrofti.* Infection and Immunity. 62:1901-1908. (46)

Semnani RT, Law M, Kubofcik N, Nutman TB. 2004. Filaria-induced immune evasion: Suppression by the infective stage of *Brugia malayi* at the earliest host-parasite interface. The Journal of Immunology. 172:6229-6238. (45)

Kazura JW. Davis RS. 1982. Soluble *Brugia malayi* microfilarial antigens protect mice against challenge by an antibody-dependent mechanism. The Journal of Immunology. 128:1792-1796. (42)

Weil GJ, Liftis F. 1987. Identification and partial characterization of a parasite antigen in sera from humans infected with *Wuchereria bancrofti.* The Journal of Immunology. 138:3035-3041. (41)

Lammie PJ, Katz SP. 1983. Immunoregulation in experimental filariasis. II. Responses to parasite and nonparasite antigens in jirds with *Brugia pahangi*. The Journal of Immunology.

130:1386-1389. (41)

Hall LR, Mehlotra RK, Higgins AW, Haxhiu MA, Pearlman E. 1998. An essential role for interleukin-5 and eosinophils in helminth-induced airway hyperresponsiveness. Infection and Immunity. 66:4425-4430. (38)

Semnani RT, Liu AY, Sabzevari H, Kubofcik J, Zhou J, Gilden JK, Nutman TB. 2003. *Brugia malayi* microfilariae induce cell death in human dendritic cells, inhibit their ability to make IL-

12 and IL-10, and reduce their capacity to activate CD4+Tcells. The Journal of Immunology.

171:1950-1960. (37)

Semnani RT, Sabzevari H, Iyer R, Nutman TB. 2001. Filarial antigens impair the function of human dendritic cells during differentiation. Infection and Immunity. 69:5813-5822. (36)

Gottsch JD, Eisinger SW, Liu SH, Scott AL. 1999. Calgranulin C has filariacidal and filariastatic activity. Infection and Immunity. 67:6631-6636. (35)

Pearlman E, Kroeze WK, Hazlett FE, Chen SSA, Mawhorter SD, Boom WH, Kazura JW. 1993. *Brugia malayi*: Acquired resistance to microfilariae in BALB/C mice correlates with local Th2 responses. Experimental Parasitology. 76:200-208. (34)

Pearlman E, Heinzel FP, Hazlett FE Jr, Kazura JW. 1995. IL-12 modulation of T helper responses to the filarial helminth, *Brugia malayi.* The Journal of Immunology. 154:4658-4664. (34)

Canlas M, Wadee A, Lamontagne L, Piessens WF. 1984. A monoclonal antibody to surface antigens on microfilariae of *Brugia malayi* reduces microfilaremia in infected jirds. American Journal of Tropical Medicine and Hygiene. 33:420-424. (34)

Fanning MM, Kazura JW. 1983. Genetic association of murine susceptibility to *Brugia malayi*

microfilaremia. Parasite Immunology. 5:305-316. (32)

Nutman TB, Steele C, Ward DJ, Zea-Flores G, Ottesen EA. 1991. Immunity to onchocerciasis: recognition of larval antigens by humans putatively immune to *Onchocerca volvulus* infection. Journal of Infectious Diseases. 163:1128-1133. (30)

Wamae CN, Roberts JM, Eberhard ML, Lammie PJ. 1992. Kinetics of circulating human IgG4 after diethylcarbamazine and ivermectin treatment of bancroftian filariasis. Journal of Infectious Diseases. 165:1158-1160. (30)

Gusmao RD, Stanley AM, Ottesen EA. 1981. *Brugia pahangi*: immunologic evaluation of the differential susceptibility of filarial infection in inbred Lewis rats. Experimental Parasitology.

52:147-159. (30)

Babu S, Nutman TB. 2003. Proinflammatory cytokines dominate the early immune response to filarial parasites. The Journal of Immunology. 171:6723-6732. (27)

Hussain R, Ottesen EA. 1985. IgE responses in human filariasis. III. Specificities of IgE and IgG

antibodies compared by immunoblot analysis. The Journal of Immunology. 135:1415-1420. (27)

Wamae CN, Gatika SM, Roberts JM, Lammie PJ. 1998. *Wuchereria bancrofti* in Kwale District, Coastal Kenya: patterns of focal distribution of infection, clinical manifestations and anti-filarial IgG responsiveness. Parasitology. 116:173-182. (26)

Jeffers GW, Klei TR, Enright FM, Henk WG. 1987. The granulomatous inflammatory response in jirds, *Meriones unguiculatus*, to *Brugia pahangi*: an ultrastructural and histochemical comparison of the reaction in the lymphatics and peritoneal cavity. Journal of Parasitology.

73:1220-1233. (24)

Liu LX, Weller PF. 1992. Intravascular filarial parasites inhibit platelet aggregation: Role of parasite-derived prostanoids. Journal of Clinical Investigation. 89:1113-1120. (23)

Kron M, Marquard K, Häirtlein M, Price S, Leberman R. 1995. An immunodominant antigen of *Brugia malayi* is an asparaginyl-tRNA synthetase. Federation of European Biochemical Societies Letters. 374:122-124. (21)

Wenger JD, Forsyth KP**,** Kazura JW. 1988. Identification of phosphorylcholine epitope- containing antigens in *Brugia Malayi* and relation of serum epitope levels to enfection status of jirds with Brugian filariasis. American Journal of Tropical Medicine and Hygiene. 38:133-141. (19)

Hise AG, Daehnel K, Gillette-Ferguson I, Cho E, McGarry HF, Taylor MJ, Golenbock DT, Fitzgerald KA, Kazura JW, Pearlman E. 2007. Innate immune responses to endosymbiotic *Wolbachia* bacteria in *Brugia malayi* and *Onchocerca volvulus* are dependent on TLR2, TLR6, MyD88, and Mal, but not TLR4, TRIF, or TRAM. The Journal of Immunology. 178:1068-1076. (18)

Kazura JW, Cicirello H, Forsyth K. 1986. Differential recognition of a protective filarial antigen by antibodies from humans with Bancroftian filariasis. Journal of Clinical Investigation.

77:1985-1992. (18)

Werner G, Higashi GI, Yates JA, Rajan TV. 1989. Differential recognition of two cloned *Brugia malayi* antigens by antibody class. Molecular and Biochemical Parasitology. 35:209-218. (18)

Semnani RT, Venugopal PG, Leifer CA, Mostböck S, Sabzevari H, Nutman TB. 2008. Inhibition of TLR3 and TLR4 function and expression in human dendritic cells by helminth parasites. Blood. 112:1290-1298. (17)

Ramalingam T, Rajan B, Lee J, Rajan TV. 2003. Kinetics of Cellular Responses to intraperitoneal *Brugia pahangi* infections in normal and immunodeficient mice. Infection and Immunity. 71:4361-4367. (15)

Grove DI, Davis RS, Warren KS. 1979. *Brugia malayi* microfilaremia in mice: A model for the study of the host response to microfilariae. Parasitology. 79:303-316. (15)

Weil GJ, Kumar H, Santhanam S, Sethumadhavan KVP, Jain DC. 1986. Detection of circulating parasite antigen in bancroftian filariasis by counterimmunoelectrophoresis. American Journal of Tropical Medicine and Hygiene. 35:565-570. (14)

Babu S, Blauvelt CP, Nutman TB. 2007. Filarial parasites induce NK cell activation, type 1 and type 2 cytokine secretion, and subsequent apoptotic cell death. The Journal of Immunology.

179:2445-2456. (14)

Lammie PJ, Katz SP. 1984. Immunoregulation in experimental filariasis. III. Demonstration and characterization of antigen-specific suppressor cells in the spleen of *Brugia pahangi*-infected jirds. Immunology. 52:211-219. (14)

Babu S, Kumaraswami V, Nutman TB. 2005. Transcriptional control of impaired Th1 responses in patent lymphatic filariasis by T-Box expressed in T cells and suppressor of cytokine signaling genes. Infection and Immunity. 73:3394-3401. (14)

Rajan TV, Nelson K, Shultz LD, Shultz KL, Beamer WG, Yates J, Greiner DL. 1994. Influence of gonadal steroids on susceptibility to *Brugia malayi* in scid mice. Acta tropica. 56:307-314. (14)

Weil GJ, Powers KG, Parbuoni EL, Line BR, Furrow RD, Ottesen EA. 1982. *Dirofilaria immitis*

VI. Antimicrofilarial immunity in experimental filariasis. American Journal of Tropical

Medicine and Hygiene. 31:477-485. (13)

Lal RB, Ottesen EA. 1988. Characterization of stage-specific antigens of infective larvae of the filarial parasite *Brugia malayi.* The Journal of Immunology. 140:2032-2038. (12)

Bailey JW, Hightower AW, Eberhard ML, Lammie PJ. 1995. Acquisition and expression of humoral reactivity to antigens of infective stages of filarial larvae. Parasite Immunology. 17:617-

623. (12)

Spencer L, Shultz L, Rajan TV. 2001. Interleukin-4 receptor–stat6 signaling in murine infections with a tissue-dwelling nematode parasite. Infection and Immunity. 69:7743-7752. (12)

Daehnel K, Gillette-Ferguson I, Hise AG, Diaconu E, Harling MJ, Heinzel FP, Pearlman E. 2007. Filaria/*Wolbachia* activation of dendritic cells and development of Th1-associated responses is dependent on Toll-like receptor 2 in a mouse model of ocular onchocerciasis (river blindness). Parasite Immunology. 29:455-465. (11)

Paciorkowski N, Shultz LD, Rajan TV. 2003. Primed peritoneal B lymphocytes are sufficient to transfer protection against *Brugia pahangi* infection in mice. Infection and Immunity. 71:1370-

1378. (11)

Rajan TV, Nelson FK, Shultz LD, Koller BH, Greiner DL. 1992. CD8+ T lymphocytes are not required for murine resistance to human filarial parasites. Journal of Parasitology. 78:744-746. (11)

Rao UR, Zometa CS, Vickery AC, Kwa BH, Nayar JK, Sutton ET. 1996. Effect of *Brugia malayi* on the growth and proliferation of endothelial cells *in vitro*. Journal of Parasitology.

82:550-556. (10)

Leiva LE, Lammie PJ. 1989. Regulation of parasite antigen-induced T cell growth factor activity and proliferative responsiveness in *Brugia pahangi* infected jirds. The Journal of Immunology.

142:1304-1309. (9)

Mehlotra RK, Hall LR, Higgins AW, Dreshaj IA, Haxhiu MA, Kazura JW, Pearlman E. 1998. Interleukin-12 suppresses filaria-induced pulmonary eosinophilia, deposition of major basic protein and airway hyperresponsiveness. Parasite Immunology. 20:455-462. (9)

Dissanayake S, Perler FB, Xu M, Southworth MW, Yee CK, Wang S, Dreyer G, Watawana L, Kurniawan L, Fuhrman JA, Piessens WF. 1995. Differential recognition of microfilarial chitinase, a transmission-blocking vaccine candidate antigen, by sera from patients with Brugian and Bancroftian filariasis. American Journal of Tropical Medicine and Hygiene. 53:289-294. (8)

Kazura JW, Hazlett FE Jr, Pearlman E, Day K, el-Zeiny A, Nilsen TW, Alpers MP. 1992. Antigenicity of a protective recombinant filarial protein in human bancroftian filariasis. Journal of Infectious Diseases. 166:1453-1457. (8)

Babu S, Blauvelt CP, Kumaraswami V, Nutman TB. 2005. Chemokine receptors of T cells and of B cells in lymphatic filarial infection: A role for CCR9 in pathogenesis. Journal of Infectious Diseases. 191:1018-1026. (8)

Hitch WL, Lammie PJ, Walker EM, Hightower AW, Eberhard ML. 1991. Antifilarial cellular responses detected in a Haitian pediatric population by use of a microblastogenesis assay. Journal of Infectious Diseases. 164:811-813. (8)

Ramalingam T, Ganley-Leal L, Porte P, Rajan TV. 2003. Impaired clearance of primary but not secondary *Brugia* infections in IL-5 deficient mice. Experimental Parasitology. 105:131-139. (8)

Giambartolomei GH, Lasater BL, Villinger F, Dennis VA. 1998. Diminished production of T helper 1 cytokines and lack of induction of IL-2R+ T cells correlate with T-cell unresponsiveness in rhesus monkeys chronically infected with *Brugia malayi*. Experimental Parasitology. 90:77-

85. (7)

Weil GJ, Ottesen EA, Powers KG. 1981. *Dirofilaria immitis*: parasite-specific humoral and cellular immune responses in experimentally infected dogs. Experimental Parasitology. 51:80-

86. (7)

Hussain R, Kaushal NA, Ottesen EA. 1985. Comparison of immunoblot and immunoprecipitation methods for analyzing cross-reactive antibodies to filarial antigens. Journal of Immunological Methods. 84:291-301. (7)

Prier RC, Lammie PJ. 1988. Differential regulation of *in vitro* humoral and cellular immune responsiveness in *Brugia pahangi*-infected jirds. Infection and Immunity. 56:3052-3057. (7)

Porksakorn C, Nuchprayoon S, Park K, Scott AL. 2007. Proinflammatory cytokine gene expression by murine macrophages in response to *Brugia malayi Wolbachia* surface protein. Mediators of Inflammation. 2007:84318. (6)

Ramalingam T, Porte P, Lee J, Rajan TV. 2005. Eosinophils, but not eosinophil peroxidase or major basic protein, are important for host protection in experimental *Brugia pahangi* infection. Infection and Immunity. 73:8442-8443. (6)

Talaat KR. Bonawitz RE, Domenech P, Nutman TB. 2006. Preexposure to live *Brugia malayi* microfilariae alters the innate response of human dendritic cells to *Mycobacterium tuberculosis*. The Journal of Infectious Diseases. 193:196-204. (6)

Semnani RT, Venugopal PG, Mahapatra L, Skinner JA, Meylan F, Chien D, Dorward DW, Chaussabel D, Siegel RM, Nutman TB. 2008. Induction of TRAIL- and TNF-alpha-dependent apoptosis in human monocyte-derived dendritic cells by microfilariae of *Brugia malayi.* The Journal of Immunology. 181:7081-7089. (6)

Leiva LE, Lammie PJ. 1989. Modulation of lymphocyte activation by soluble *Brugia pahangi*

extracts. Tropical Medicine and Parasitology. 40:327-331. (5)

Lammie PJ, Katz SP. 1984. Immunoregulation in experimental filariasis. IV. Induction of non- specific suppression following *in vitro* exposure of spleen cells from infected jirds to *Brugia pahangi* antigen. Immunology. 52:221-229. (5)

Michaud LA, Lammie PJ. 1988. Regulation of jird lymphocyte responsiveness to fractionated antigens of *Brugia pahangi*. Tropical Medicine and Parasitology. 39:317-321. (4)

Veerapathran A, Dakshinamoorthy G, Gnanasekar M, Reddy MVR, Kalyanasundaram R. 2009. Evaluation of *Wuchereria bancrofti* GST as a vaccine candidate for lymphatic filariasis. PLoS Neglected Tropical Diseases. 3:e457. (4)

Mehlotra RK, Hall LR, Haxhiu MA, Pearlman E. 2001. Reciprocal immunomodulatory effects of gamma interferon and interleukin-4 on filaria-induced airway hyperresponsiveness. Infection and Immunity. 69:1463-1468. (4)

Smith HL, Rajan TV. 2001. Inhibitors of the lipoxygenase pathway block development of *Brugia malayi* L3 *in vitro*. Journal of Parasitology. 87:242-249. (4)

Lammie PJ, Katx SP. 1983. Immunoregulation in experimental filariasis. I. *In vitro* suppression of mitogen-induced blastogenesis by adherent cells from jirds chronically infected with *Brugia pahangi.* The Journal of Immunology. 130:1381-1385. (3)

Kron MA, Cichanowicz S, Hendrick A, Liu A, Leykan J, Kuhn LA. 2008. Using structural analysis to generate parasite-selective monoclonal antibodies. Protein Science. 17:983-989. (2)

Gnanasekar M, Padmavathi B, Ramaswamy K. 2005. Cloning and characterization of a novel immunogenic protein 3 (NIP3) from *Brugia malayi* by immuno screening of a phage-display cDNA expression library. Parasitology Research. 97:49-58. (2)

Chenthamarakshan V, Cheirmaraj K, Reddy MVR, Harinath BC. 1997. Immunoprophylactic studies with a 43 kDa human circulating filarial antigen and a cross reactive 120 kDa *Brugia malayi* sodium dodecyl sulphate soluble antigen in filariasis. Journal of Biosciences*.* 22:91-98. (2)

Thompson JP, Crandall RB, Doyle TJ IV, Hines SA, Crandall CA. 1986. Antibody and cellular immune responses to microfilarial antigens in ferrets experimentally infected with *Brugia malayi*. Zeitschrift für parasitenkunde. 72:525-535. (2)

Shenoy RK, Rakesh PG, Baldwin CI, DA. 1996. The sheath of the microfilaria of *Brugia malayi*

from human infections has IgG on its surface. Parasitology Research. 82:382-384. (1)

Fanning MM, Kazura JW. 1986. Lack of biological significance of *in vitro Brugia malayi* microfilarial cytotoxicity mediated by *Propionibacterium acnes* ("*Corynebacterium parvum*")- and *Mycobacterium bovis* BCG-activated macrophages. Infection and Immunity. 52:534-537. (1)

Bennuru S, Nutman TB. 2009. Lymphangiogenesis and lymphatic remodeling induced by filarial parasites: Implications for pathogenesis. PLoS Pathogens 5:e1000688. (1)

Crandall R, Crandall C, Nayar J, Doyle T. 1994. Resistance and disease in *Brugia malayi* infection of ferrets following prior infection, injection of attenuated infective larvae and injections of larval extracts. Parasite Immunology. 16:425-433. (1)

Dash Y, Ramesh M, Greiner D, Shultz LD, Klei TR, Rajan TV. 2007. Determinants of memory in experimental filarial infections in mice. Parasite Immunology. 29:567-574. (1)

Giambartolomei GH, Lasater BL, Villinger F, Dennis VA. 2001. Diminished expression of the costimulatory ligand CD80 (B7.1) gene correlates with antigen-specific cellular unresponsiveness in rhesus monkeys with lymphatic filariasis. Acta Tropica. 78:67-71. (1)

Rajan TV, Bailis JM, Yates JA, Shultz LD, Greiner DL, Nelson FK. 1994. Maternal influence on susceptibility of offspring to *Brugia malayi* infection in a murine model of filariasis. Acta Tropica. 58:283-289. (1)

Dash Y, Ramesh M, Kalyanasundaram R, Munirathinam G, Shultz LD, Rajan TV. 2011. Granuloma formation around *Brugia* larvae triggered by host responses to an E/S antigen. Infection and Immunity. 79:838-845. (0)

Samykutty A, Dakshinamoorthy G, Kalyanasundaram R. 2010. Multivalent vaccine for lymphatic filariasis. Procedia in Vaccinology. 3:12-18. (0)

Patel A, Chojnowski AN, Gaskill K, De Martini W, Goldberg RL, Siekierka JJ. 2010. The role of a *Brugia malayi* p38 MAP kinase ortholog (Bm-MPK1) in parasite anti-oxidative stress responses. Molecular and Biochemical Parasitology. Article in print. (0)

Cho-Ngwa F, Liu J, Lustigman S. 2010. The *Onchocerca volvulus* cysteine proteinase inhibitor, *Ov*-CPI-2, as a target of protective antibody response that increases with age. PLoS Neglected Tropical Diseases. 4:e800. (0)

Semnani RT, Mahapatra L, Dembele B, Konate S, Metenou S, Dolo H, Coulibaly ME, Soumaoro L, Coulibaly SY, Sanogo D, Seriba Doumbia S, Diallo AA, Traoré SF, Klion A,

Nutman TB, Mahanty S. 2010. Expanded numbers of circulating myeloid dendritic cells in patent human filarial infection reflect lower CCR1 expression. The Journal of Immunology.

185:6364-6372. (0)

Weinkopff T, Atwood JA III, Punkosdy GA, Moss D, Weatherly DB, Orlando R, Patrick Lammie P. 2009. Identification of antigenic *Brugia* adult worm proteins by peptide mass fingerprinting. Journal of Parasitology. 95:1429-1435. (0)

Ellenberger DL, Lammie PJ. 1992. Immunologic characterization of jird lymphocyte responsiveness to *Brugia pahangi* ribosomal protein S13. Experimental Parasitology. 75:293-

302. (0)

Kalyanasundaram R, Balumuri P. 2011. Multivalent vaccine formulation with BmVAL-1 and BmALT-2 confer significant protection against challenge infections with *Brugia malayi* in mice and jirds. Research and Reports in Tropical Medicine. 2:45-56. (0)

Ramesh M, Paciorkowski N, Dash Y, Schultz L, Rajan TV. 2007. Acute but not chronic macrophage recruitment in filarial infections in mice is dependent on C-C chemokine ligand 2. Parasite Immunology. 29:395-404.

**Molecular**

Blaxter ML, Raghavan N, Ghosh I, Guiliano D, Lu W, Williams SA. Slatko B, Scott AL. 1996. Genes expressed in *Brugia malayi* infective third stage larvae. Molecular and Biochemical Parasitology. 77:77-93. (97)

Gregory WF, Blaxter ML, Maizels RM. 1997. Differentially expressed, abundant trans-spliced cDNAs from larval *Brugia malayi*. Molecular and Biochemical Parasitology. 87:85-95. (53)

Chandrashekar R, Tsuji N, Morales T, Ozols V, Mehta K. 1998. An ERp60-like protein from the filarial parasite *Dirofilaria immitis* has both transglutaminase and protein disulfide isomerase activity. Proceedings of the National Academy of Sciences of the United States of America.

95:531-536. (51)

Yenbutr P, Scott AL. 1995. Molecular cloning of a serine proteinase inhibitor from *Brugia malayi.* Infection and Immunity. 63:1745-1753. (51)

Gnanasekar M, Rao KV, Chen L, Narayanan RB, Geetha M, Scott AL, Ramaswamy K, Kaliraj

P. 2002. Molecular characterization of a calcium binding translationally controlled tumor protein homologue from the filarial parasites *Brugia malayi* and *Wuchereria bancrofti*. Molecular and Biochemical Parasitology. 121:107-118. (44)

Kaushal NA, Hussain R, Nash TE, Ottesen EA. 1982. Identification and characterization of excretory-secretory products of *Brugia malayi,* adult filarial parasites. The Journal of Immunology. 129:338-343. (40)

Ghosh I, Eisinger SW, Raghavan N, Scott AL. 1998. Thioredoxin peroxidases from *Brugia malayi*. Molecular and Biochemical Parasitology. 91:207-220. (40)

Venegas A, Goldstein JC, Beauregard K, Oles A, Abdulhayoglu N, Fuhrman JA. 1996. Expression of recombinant microfilarial chitinase and analysis of domain function. Molecular and Biochemical Parasitology. 78:149-159. (26)

Higazi TB, Merriweather A, Shu L, Davis R, Unnasch TR. 2002. *Brugia malayi:* Transient transfection by microinjection and particle bombardment. Experimental Parasitology. 100:95-

102. (26)

Moreno Y, Geary TG, 2008. Stage- and gender-specific proteomic analysis of *Brugia malayi*

excretory-secretory products. PLoS Neglected Tropical Diseases. 2:e326. (23)

Li B-W, Rush AC, Tan J, Weil GJ. 2004. Quantitative analysis of gender-regulated transcripts in the filarial nematode *Brugia malayi* by real-time RT-PCR. Molecular and Biochemical Parasitology. 137:329-337. (22)

Shu L, Katholi CR, Higazi T, Unnasch TR. 2003. Analysis of the *Brugia malayi* HSP70 promoter using a homologous transient transfection system. Molecular and Biochemical Parasitology. 128:67-75. (22)

Bennuru S, Semnani R, Meng Z, Ribeiro JMC, Veenstra TD, Nutman TB. 2009. *Brugia malayi* excreted/secreted proteins at the host/parasite interface: Stage- and gender-specific proteomic profiling. PLoS Neglected Tropical Diseases 3:e410. (21)

Li BW, Rush AC, Crosby SD, Warren WC, Williams SA, Mitreva M, Weil GJ. 2005. Profiling of gender-regulated gene transcripts in the filarial nematode *Brugia malayi* by cDNA oligonucleotide array analysis. Molecular and Biochemical Parasitology. 143:49–57. (20)

Harris MT, Lai K, Arnold K, Martinez HF, Specht CA, Fuhrman JA. 2000. Chitin synthase in the filarial parasite, *Brugia malayi.* Molecular and Biochemical Parasitology. 111:351-362. (19)

Guenette S, Prichard RK, Klein RD, Matlashewski G. 1991. Characterization of ß-tubulin gene and ß-tubulin gene products of *Brugia pahangi.* Molecular and Biochemical Parasitology.

44:153-164. (19)

Kaushal NA, Simpson AJG, Hussain R, Ottesen EA. 1984. *Brugia malayi:* Stage-specific expression of carbohydrates containing *N*-acetyl-d-glucosamine on the sheathed surfaces of microfilariae. Experimental Parasitology. 58:182-187. (19)

Michalski ML, Weil GJ. 1999. Gender-specific gene expression in *Brugia malayi*. Molecular and

Biochemical Parasitology. 104:247-257. (17)

Fuhrman JA, Lee J, Dalamagas D. 1995. Structure and function of a family of chitinase isozymes from Brugian microfilariae. Experimental Parasitology. 80:672-680. (17)

Rothstein N, Stoller TJ, Rajan TV. 1988. DNA base composition of filarial nematodes. Parasitology. 97:75-79. (15)

Raghavan N, Ghosh I, Eisinger WS, Pastrana D, Scott AL. 1999. Developmentally regulated expression of a unique small heat shock protein in *Brugia malayi*. Molecular and Biochemical Parasitology. 104:233-246. (15)

Yates DM, Wolstenholme AJ. 2004. An ivermectin-sensitive glutamate-gated chloride channel subunit from *Dirofilaria immitis*. International Journal for Parasitology. 34:1075-1081. (14)

Merriweather A, Guenzler V, Brenner M, Unnasch TR. 2001. Characterization and expression of enzymatically active recombinant filarial prolyl 4-hydroxylase. Molecular and Biochemical Parasitology. 116:185-197. (14)

Pfarr KM, Fuhrman JA. 2000. *Brugia malayi:* localization of nitric oxide synthase in a lymphatic filariid. Experimental Parasitology. 94:92-98. (12)

Pfarr KM, Qazi S, Fuhrman JA. 2001. Nitric oxide synthase in filariae: Demonstration of nitric oxide production by embryos in *Brugia malayi* and *Acanthocheilonema viteae.* Experimental Parasitology. 97:205-214. (12)

Kaiser L, Geary TG, Williams JF. 1998. *Dirofilaria immitis* and *Brugia pahangi:* Filarial parasites make nitric oxide. Experimental Parasitology. 90:131-134. (12)

Longworth DL, Monahan-Earley RA, Dvorak AM, Weller PF. 1988. *Brugia malayi*: Arachidonic acid uptake into lipid bodies of adult parasites. Experimental Parasitology. 65:251-

257. (11)

Ravi V, Kubofcik J, Bandopathyaya S, Geetha M, Narayanan RB, Nutman TB, Kaliraj P. 2004. *Wuchereria bancrofti:* cloning and characterization of heat shock protein 70 from the human lymphatic filarial parasite. Experimental Parasitology.106:1-10. (11)

Schmitz KA, Hale TJ, Rajan TV, Yates JA. 1996. Localization of paramyosin, myosin, and a heat shock protein 70 in larval and adult *Brugia malayi*. Journal of Parasitology. 82:367-370. (11)

Ford L, Zhang J, Liu J, Hashmi S, Fuhrman JA, Oksov Y, Lustigman S. 2009. Functional analysis of the cathepsin-like cysteine protease genes in adult *Brugia malayi* using RNA interference. PLoS Neglected Tropical Diseases. 3:e377. (11)

Liu LX, Weller PF. 1989. *Brugia malayi*: Microfilarial polyunsaturated fatty acid composition and synthesis. Experimental Parasitology. 69:198-203. (10)

Borchert N, Becker-Pauly C, Wagner A, Fischer P, Stöcker W, Brattig NW.2007. Identification and characterization of onchoastacin, an astacin-like metalloproteinase from the filaria *Onchocerca volvulus.* Microbes and Infection. 9:498-506. (10)

Kron M, Petridis M, Milev Y, Leykam J, Hartlein M. 2003. Expression, localization and alternative function of cytoplasmic asparaginyl-tRNA synthetase in *Brugia malayi.* Molecular and Biochemical Parasitology. 129:33-39. (9)

Arnold K, Venegas A, Houseweart C, Fuhrman JA. 1996. Discrete transcripts encode multiple chitinase isoforms in Brugian microfilariae. Molecular and Biochemical Parasitology. 80:149-

158. (9)

Wolff KM, Scott AL. 1995. *Brugia malayi*: Retinoic acid uptake and localization. Experimental

Parasitology. 80:282-290. (9)

Higazi TB, Deoliveira A, Katholi CR, Shu L, Barchue J, Lisanby M, Unnasch TR. 2005. Identification of elements essential for transcription in *Brugia malayi* promoters. Journal of Molecular Biology. 353:1-13. (9)

Ghosh I, Raghavan N, FitzGerald PC, Scott AL. 1995. Nucleoside diphosphate kinase from the parasitic nematode *Brugia malayi.* Gene. 164:261-266. (8)

Moroz OV, Blagova EV, Wilkinson AJ, Wilson KS, Bronstein IB. 2009. The crystal structures of human S100A12 in apo form and in complex with zinc: New insights into S100A12 oligomerisation. Journal of Molecular Biology. 391:536-551. (7)

Tang L, Prichard RK. 1989. Characterization of tubulin from *Brugia malayi* and *Brugia pahangi*. Molecular and Biochemical Parasitology. 32:145-152. (6)

Triteeraprapab S, Richie TL, Tuan RS, Shelpey KJ, Dinman JD, Neubert TA, Scott AL. 1995. Molecular cloning of a gene expressed during early embryonic development in *Onchocerca volvulus*. Molecular and Biochemical Parasitology. 69:161-171. (6)

Bughio NI, Faubert GM, Prichard R. 1993. Characterization and biological activities of anti-

*Brugia pahangi* tubulin monoclonal antibodies. International Journal for Parasitology. 23:913-

924. (5)

Kadipasaoglu AK, Bilge FH. 1989. Partial characterization of the adsorbed protein layer on

*Dirofilaria immitis* (Nematoda) cuticle. Parasitology Research. 75:554-558. (5)

Jiang D, Li BW, Fischer PU, Weil GJ. 2008. Localization of gender-regulated gene expression in the filarial nematode *Brugia malayi*. International Journal for Parasitology. 38:503-512. (4)

Crossgrove K, Maina CV, Robinson-Rechavi M, Lochner MC. 2008. Orthologues of the *Drosophila melanogaster E75* molting control gene in the filarial parasites *Brugia malayi* and *Dirofilaria immitis.* Molecular and Biochemical Parasitology. 157:92-97. (4)

Guenette S, Prichard RK, Matlashewski G. 1992. Identification of a novel *Brugia pahangi* ß- tubulin gene (ß2) and a 22-nucleotide spliced leader sequence on ß1-tubulin mRNA. Molecular and Biochemical Parasitology. 50:275-284. (2)

Kron M, Leykam J, Kopaczewski J, Matus I. 2007. Identification of diadenosine triphosphate in *Brugia malayi* by reverse phase high performance liquid chromatography and MALDI mass spectrometry. Journal of Chromatography. B, Analytical Technologies in the Biomedical and Life Sciences. 856:234-238. (2)

Ardelli BF, Stitt LE, Tompkins JB. 2010. Inventory and analysis of ATP-binding cassette (ABC)

systems in *Brugia malayi*. Parasitology. 137:1195-1212. (1)

McNulty SN, Weil GJ, Heinz M, Crosby SD, Fischer PU. 2008. *Brugia malayi:* Whole genome amplification for genomic characterization of filarial parasites. Experimental Parasitology.

119:256-263. (0)

Kariuki MM, Hearne LB, Beerntsen BT. 2010. Differential transcript expression between the microfilariae of the filarial nematodes, *Brugia malayi* and *B. pahangi*. BioMed Central Genomics*.* 11:225. (0)

Williams SA. 2004. 3.3 Filarial genomics. American Journal of Tropical Medicine and Hygiene.

71:37-40. (0)

Li BW, Rush AC, Jiang DJ, Mitreva M, Abubucker S, Weil GJ. 2011. Gender-associated genes in filarial nematodes are important for reproduction and potential intervention targets. PLoS Neglected Tropical Diseases. 5:e947. (0)

Song C, Gallup JM, Day TA, Bartholomay LC, Kimber MJ. 2010. Development of an *in vivo* RNAi protocol to investigate gene function in the filarial nematode, *Brugia malayi*. PLoS Pathogens. 6:e1001239. (0)

Bourguinat C, Keller K, Prichard RK, Geary TG. 2011. Genetic polymorphism in *Dirofilaria immitis*. Veterinary Parasitology. 176:368-373. (0)

Xu S, Liu C, Tzertzinis G, Ghedin E, Evans CC, Kaplan R, Unnasch TR. 2011. *In vivo* transfection of developmentally competent *Brugia malayi* infective larvae. International Journal for Parasitology. Article in Press. (0)

Bailey M, Chauhan C, Liu Cand, Unnasch TR. 2011. The role of polymorphisms in the spliced leader addition domain in determining promoter activity in *Brugia malayi.* Molecular and Biochemical Parasitology. 176:37-41. (0)

Amambua AN, Ghogomu SM, Tachu JB, Pelle R, Titanji VP. 2005. Characterization of a heparan sulfate proteoglycan homologue OvPG-1 from *Onchocerca volvulus*. East African Medical Journal. 82:614-624. (0)

**Other**

Wisely SM, Howard J, Williams SA, Bain O, Santymire RM, Bardsley KD, Williams ES. 2008. An unidentified filarial species and its impact on the fitness in wild populations of the black- footed ferret (*Mustela nigripes*). Journal of Wildlife Diseases. 44:53-64. (4)

Vande Waa EA, Foster LA, DeRuiter J, Guderian RH, Williams JF, Geary TG. 1993. Glutamine- supported motility of adult filarial parasites *in vitro* and the effect of glutamine antimetabolites. Journal of Parasitology. 79:173-180. (3)

Landmann F, Foster JM, Slatko B, Sullivan W. 2010. Asymmetric *Wolbachia* segregation during early *Brugia malayi* embryogenesis determines is distribution in adult host tissues. PLoS Neglected Tropical Diseases. 4:e758. (1)

Vaughan JA, Focks DA, Turell MJ. 2009. Simulation models examining the effect of Brugian filariasis on Dengue epidemics. American Journal of Tropical Medicine and Hygiene. 80:44-50. (0)

Michalski ML, Bain O, Fischer K, Fischer PU, Kumar S, Foster JM. 2010. Identification and phylogenetic analysis of *Dirofilaria ursi* (Nematoda: Filarioidea) from Wisconsin black bears (*Ursus americanus*) and its *Wolbachia* endosymbiont. Journal of Parasitology. 96:412–419. (0)

**Vector Biology**

Christensen BM, Sutherland DR. 1984. *Brugia pahangi*: Exsheathment and midgut penetration in *Aedes aegypti*. Transactions of the American Microscopical Society. 103:423-433. (66)

Ferdig MT, Beerntsen BT, Spray FJ, Li J, Christensen BM. 1993. Reproductive costs associated with resistance in a mosquito-filarial worm system. American Journal of Tropical Medicine and Hygiene. 49:756-762. (55)

Christensen BM, Sutherland DR, Gleason LN. 1984. Defense reactions of mosquitoes to filarial worms: Comparative studies on the response of three different mosquitoes to inoculated *Brugia pahangi* and *Dirofilaria immitis* microfilariae. Journal of Invertebrate Pathology. 44:267-274. (43)

Lowenberger CA, Ferdig MT, Bulet P, Khalili S, Hoffmann JA, Christensen BM. 1996. *Aedes aegypti:* Induced antibacterial proteins reduce the establishment and development of *Brugia malayi.* Experimental Parasitology. 83:191-201. (42)

Beerntsen BT, Christensen BM. 1990. *Dirofilaria immitis:* Effect on hemolymph polypeptide synthesis in *Aedes aegypti* during melanotic encapsulation reactions against microfilariae. Experimental Parasitology. 71:406-414. (32)

Beerntsen BT, Luckhart S, Christensen BM. 1989. *Brugia malayi* and *Brugia pahangi*: Inherent difference in immune activation in the mosquitoes *Armigeres subalbatus* and *Aedes aegypti*. Journal of Parasitology. 75:76-81. (32)

Li J, Tracy JW, Christensen BM. 1992. Phenol oxidase activity in hemolymph compartments of *Aedes aegypti* during melanotic encapsulation reactions against microfilariae. Developmental and Comparative Immunology. 16:41-48. (29)

Beerntsen BT, Severson DW, Klinkhammer JA, Kassner VA, Christensen BM. 1995. *Aedes aegypti:* A quantitative trait locus (QTL) influencing filarial worm intensity is linked to QTL for susceptibility to other mosquito-borne pathogens. Experimental Parasitology. 81:355-362. (28)

Beerntsen BT, Severson DW, Christensen BM. 1994. *Aedes aegypti*: characterization of a hemolymph polypeptide expressed during melanotic encapsulation of filarial worms. Experimental Parasitology. 79:312-321. (25)

Wattam AR, Christensen BM. 1992. Induced polypeptides associated with filarial worm refractoriness in *Aedes aegypti*. Proceedings of the National Academy of Sciences of the United States of America. 89:6502-6505. (25)

Wang X, Fuchs JF, Infanger LC, Rocheleau TA, Hillyer JF, Chen CC, Christensen BM. 2005. Mosquito innate immunity: Involvement of beta 1,3-glucan recognition protein in melanotic encapsulation immune responses in *Armigeres subalbatus*. Molecular and Biochemical Parasitology. 139:65-73. (24)

Infanger LC, Rocheleau TA, Bartholomay LC, Johnson JK, Fuchs N, Higgs S, Chen CC, Christensen BM. 2004. The role of phenylalanine hydroxylase in melanotic encapsulation of filarial worms in two species of mosquitoes. Insect Biochemistry and Molecular Biology.

34:1329-1338. (21)

Fischer P, Erickson SM, Fischer K, Fuchs JF, Rao RU, Christensen BM, Weil GJ. 2007. Persistence of *Brugia malayi* DNA in vector and non-vector mosquitoes: Implications for xenomonitoring and transmission monitoring of lymphatic filariasis. American Journal of Tropical Medicine and Hygiene. 76:502-507. (15)

Ibrahim MS, Richie TL, Scott AL. 1992. Surface-associated antigens of *Brugia malayi* L2 and

L3 parasites during vector-stage development. Molecular and Biochemical Parasitology. 52:97-

110. (15)

Vaughan JA, Turell MJ. 1996. Dual host infections: Enhanced infectivity of eastern equine encephalitis virus to *Aedes* mosquitoes mediated by *Brugia* microfilariae. American Journal of Tropical Medicine and Hygiene. 54:105-109. (13)

Guo X, Beerntsen BT, Zhao X, Christensen BM. 1995. Hemocyte alterations during melanotic encapsulation of *Brugia malayi* in the mosquito *Armigeres Subalbatus*. Journal of Parasitology.

81:200-207. (12)

Lafond MM, Christensen BM, Lasee BA. 1985. Defense reactions of mosquitoes to filarial worms: potential mechanism for avoidance of the response by *Brugia pahangi* microfilariae. Journal of Invertebrate Pathology. 46:26-30. (12)

Berry WJ, Rowley WA, Christensen BM. 1986. Influence of developing *Brugia pahangi* on spontaneous flight activity of *Aedes aegypti* (Diptera: Culicidae). Journal of Medical Entomology. 23:441-445. (10)

Christensen BM, LaFond MM. 1986. Parasite-induced suppression of the immune response in

*Aedes aegypti* by *Brugia pahangi*. Journal of Parasitology. 72:216-219. (9)

Christensen BM, Forton KF, Lafond MM, Grieve RB. 1987. Surface changes on *Brugia pahangi* microfilariae and their association with immune evasion in *Aedes aegypti*. Journal of Invertebrate Pathology. 49:14-18. (9)

Aliota MT, Fuchs JF, Mayhew GF, Chen CC, Christensen BM. 2007. Mosquito transcriptome changes and filarial worm resistance in *Armigeres subalbatus*. BioMed Central genomics. 8:463. (9)

Erickson SM, Xi Z, Mayhew GF, Ramirez JL, Aliota MT, Christensen BM, Dimopoulos G.

2009. Mosquito infection responses to developing filarial worms. PLoS Neglected Tropical

Diseases. 3:e529. (8)

Vaughan JA, Trpis M, Turell MJ. 1999. *Brugia malayi* microfilariae (Nematoda: Filaridae) enhance the infectivity of Venezuelan equine encephalitis virus to *Aedes* mosquitoes (Diptera: Culicidae). Journal of Medical Entomology. 36:758-763. (6)

Spray FJ, Christensen BM. 1991. *Aedes aegypti*: characterization of hemocyte polypeptide synthesis during wound healing and immune response to inoculated microfilariae. Experimental Parasitology. 73:481-488. (6)

Klowden MJ. 1981. Infection of *Aedes aegypti* with *Brugia pahangi* administered by enema: Results of quantitative infection and loss of infective larvae during blood feeding. Transactions of the Royal Society of Tropical Medicine and Hygiene. 75:354-358. (4)

Beckett EB. 1990. Species variation in mosquito flight-muscle damage resulting from a single filarial infection and its repercussions on a second infection. Parasitology Research. 76:606-609. (4)

Nayar JK, Mikarts LL, Knight JW, Bradley TJ. 1992. Characterization of the intracellular melanization response in *Anopheles quadrimaculatus* against subperiodic *Brugia malayi* larvae. Journal of Parasitology. 78:876-880. (2)

Aliota MT, Fuchs JF, Rocheleau TA, Clark AK, Hillyer JF, Chen CC, Christensen BM. 2010. Mosquito transcriptome profiles and filarial worm susceptibility in *Armigeres subalbatus*. PLoS Neglected Tropical Diseases. 4:e666. (2)

Beerntsen BT, Bartholomay LC, Lowery RJ. 2007. Penetration of the mosquito midgut is not required for *Brugia pahangi* microfilariae to avoid the melanotic encapsulation response of *Armigeres subalbatus*. Veterinary Parasitology. 144:371-374. (2)

Jaffe JJ, Chrin LR. 1979. Thymidylate synthetase activity in normal and *Brugia pahangi*-infected

*Aedes aegypti*. Biochemical Pharmacology. 28:1831-1835. (1)

Griffiths KG, Mayhew GF, Zink RL, Erickson SM, Fuchs JF, McDermott CM, Christensen BM, Michalski ML. 2009. Use of microarray hybridization to identify *Brugia* genes involved in mosquito infectivity. Parasitology Research. 106:227-235. (1)

Michalski ML, Erickson SM, Bartholomay LC, Christensen BM. 2010. Midgut barrier imparts selective resistance to filarial worm infection in *Culex pipiens pipiens*. PLoS Neglected Tropical Diseases. 4:e875. (0)

Beerntsen BT, Lowery RJ. 2007. Response of *Armigeres subalbatus* (Diptera: Culicidae) to intraperitoneally isolated *Brugia* spp. microfilariae. Journal of Medical Entomology. 44:295-

298. (0)

Aliota MT, Chen CC, Dagoro H, Fuchs JF, Christensen BM. 2011. Filarial worms reduce plasmodium infectivity in mosquitoes. PLoS Neglected Tropical Diseases. 5:e963. (0)
